# Association between higher triglyceride glucose index and increased risk of osteoarthritis: data from NHANES 2015–2020

**DOI:** 10.1186/s12889-024-18272-9

**Published:** 2024-03-11

**Authors:** Jie Huang, Rigbat Rozi, Jingbo Ma, Bensheng Fu, Zhengcao Lu, Jiang Liu, Yu Ding

**Affiliations:** 1 Orthopedics of TCM Senior Department, The Sixth Medical Center of PLA General Hospital, 100048 Beijing, China; 2 Department of Orthopaedics, School of Medicine, South China University of Technology, 510006 Guangzhou, China; China; 3 Department of Orthopaedics, School of Medicine, Jinzhou Medical University, 121001 Jinzhou, China

**Keywords:** Triglyceride-glucose index, Osteoarthritis, Insulin resistance, National health and nutrition examination survey

## Abstract

**Background:**

The relationship between the triglyceride glucose (TyG) index and osteoarthritis (OA) remains unclear. The objective of this study was to examine potential associations between an elevated TyG index and an increased risk of OA prevalence.

**Methods:**

3,921 participants with OA from the National Health and Nutrition Examination Survey (2015–2020) were included in this study. Participants were categorized into quartiles based on TyG index, which was determined using the formula: Ln [triglyceride (mg/dL) fasting blood glucose (mg/dL)/2]. Weighted multivariable regression, subgroup analyses, and threshold effect analyses were performed to calculate the independent association between TyG index and OA.

**Results:**

A total of 25,514 people were enrolled, with a mean TyG index of 8.48 ± 0.65. The results of multivariable logistic regression analysis after full adjustment showed a significant association between higher TyG index values and an increased risk of OA. Specifically, each incremental unit increase in the TyG index was associated with a 634% higher risk of OA [OR = 7.34; 95% CI: 2.25, 23.93; *p* = 0.0010]. Based on interaction tests, age, gender, BMI, and smoking status did not significantly affect the relationship between the TyG index and OA, while diabetes showed a stronger positive correlation between the TyG index and OA.

**Conclusion:**

An increased risk of OA was associated with a higher TyG index. TyG could be a valuable predictor of OA and offer novel perspectives on the assessment and treatment of OA.

## Introduction

Osteoarthritis (OA) is a prevalent cause of impairment and a substantial factor in the financial burden experienced by older individuals [1–3]. The prevalence of this illness has experienced a significant increase in recent decades, primarily due to the demographic shift towards an aging population and the escalating rate of obesity [4]. The economic impact of OA in several affluent countries has been estimated to account for approximately 1–2.5% of their gross domestic product [5]. Compared with 2020, there is an anticipated substantial increase in the prevalence of OA projected for the year 2050. The projected increase in prevalence rates for different types of OA are as follows: knee OA (KOA) is estimated to increase by 74.9% (with a range of 59.4–89.9), hand OA by 48.6% (with a range of 35.9–67.1), hip OA by 78.6% (with a range of 57.7-105.3), and other forms of OA by 95.1% (with a range of 68.1–135.0) [6]. Hence, it is vital to expeditiously recognize and intervene in instances of OA in order to mitigate its prevalence.

Insulin resistance (IR) is a prevalent feature observed in persons diagnosed with type 2 diabetes mellitus (T2DM), hypertension, dyslipidemia, and cardiovascular disease. It denotes a reduced responsiveness to the physiological impacts of insulin. Numerous observational studies have provided strong evidence indicating a noteworthy correlation between diabetes and OA, with the latter ascribed to insulin resistance [7]. The TyG index is derived through a mathematical computation involving the fasting triglyceride and glucose values’ logarithm. The indicator of IR, which is a precursor to T2DM, has been universally acknowledged as a distinct and reliable measure [8]. The evidence suggests a correlation between the TyG index and the prognosis of several illnesses, such as cardiovascular disease, kidney stones, erectile dysfunction, and dementia [9–13].

Accumulating evidence suggests that IR may increase the risk of developing OA. In a study conducted by Martine Duclos et al. [14], it was observed that elevated blood glucose levels could lead to inflammation and cartilage degeneration through oxidative stress, accumulation of inflammatory mediators, and advanced glycation end-products. Florent Eymard et al. [15] suggested that T2DM prediction decreased joint space in men with established KOA. Additionally, it was noted by Daisuke Hamada et al. [16] T2DM could increase vulnerability to the beginning and development of OA by encouraging IR expression. Moreover, Zaharia et al. [17] demonstrated that IR is connected to both musculoskeletal function and symptoms of arthritis in both T2MD patients and metabolically healthy people. In addition, Bradley et al. [18] showed that leptin and the interleukin-1 receptor antagonist were connected to IR and OA strengthened the case for a link between T2DM and OA. Thus, there may be a connection between the TyG index and OA because it has been suggested that the TyG index is an IR marker. However, the correlation between TyG index and OA has never been examined in a prior study.

It is conceivable to speculate about a potential correlation between the TyG index and the occurrence of OA, given the claim that the TyG index serves as an indicator of IR. However, more investigation is required into the relationship between the TyG index and the prevalence of OA. Therefore, this study aims to examine the relationship between the TyG index and OA in a sizable, nationally representative cohort of Americans. The National Health and Nutrition Examination Survey (NHANES) from 2015 to 2020 provided the data for this investigation.

## Methods

### Study design and population

**Fig. 1:**
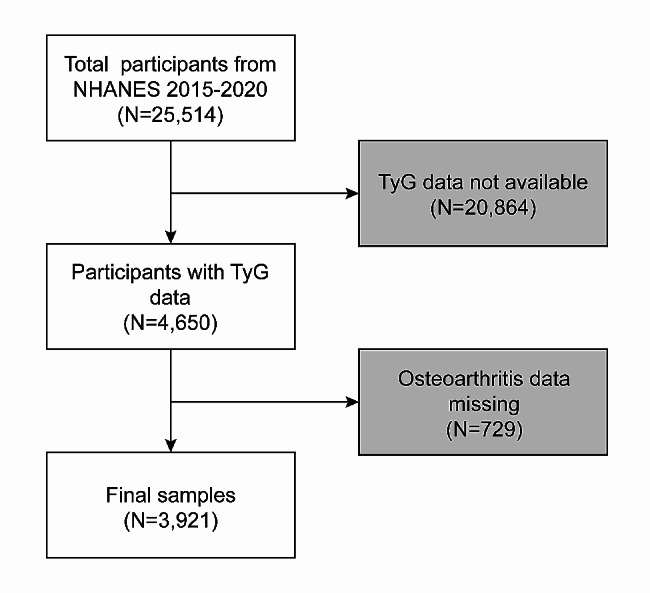
Sample selection flowchart from NHANES 2015–2020

The NAHNES is a research initiative conducted by the National Centre for Health Statistics (NCHS) aimed at gathering comprehensive health and nutrition data pertaining to the population of the United States. In order to achieve a representative sample of study participants, the organization employed a sampling methodology that incorporated stratification, multiple stages, and clustering based on probability. Additionally, prior to initiating the project, all participants had provided written informed consent for the collection of data. The information from the NHANES from 2015 to 2020 was reviewed in the current study. This analysis found that a significant fraction of the 25,514 people who were declared appropriate lacked TyG data for 20,864 people and OA data for 729 people. As seen in Fig. 1, the study ultimately enrolled 3,921 participants.

### Definitions of TyG and OA

The formula determines the TyG index: Ln [Fasting triglycerides (mg/dL) × Fasting glucose (mg/dL)/2] = TyG [19]. Data from self-reported personal interviews include information on arthritis diagnosis. Participants were asked if they had ever been informed that they had arthritis by a doctor or other medical expert. If “yes,” they were asked to categorize their arthritis diagnosis as OA, rheumatoid arthritis, psoriatic arthritis, and others. Prior research has substantiated the veracity of self-reported OA problems [20].

### Covariables

The factors that might muddle the TyG index and OA connection were summarised using the multivariable-adjusted models. Our study’s covariates included a range of demographic and health-related variables, including age (measured in years), gender (categorized as male or female), race, education level, poverty-to-income ratio (PIR), smoke status (Smoked at least 100 cigarettes in life), alcohol consumption (dichotomized as drinking at least once a month or not), hypertension, diabetes, moderate physical activity, Body Mass Index (BMI), high-Density Lipoprotein Cholesterol (HDL cholesterol), low-Density Lipoprotein Cholesterol (LDL cholesterol), insulin, homeostatic model assessment of insulin resistance (HOMA-IR), alanine transaminase (ALT), aspartate aminotransferase (AST), C-Reactive Protein (CRP), blood urea nitrogen (BUN), and uric acid (UA). The BMI was divided into three ranges: 25≤, 25–30, and ≥ 30 g/cm^2^, which correspond to persons who are average weight, overweight, and obese, respectively. The public can access the detailed measurement techniques for the research variables at www.cdc.gov/nchs/nhanes/, the official website of the Centres for Disease Control and Prevention (CDC).

### Statistical analysis

The categorization of continuous variables was determined by considering their distribution properties. The researchers computed weighted means and their corresponding standard errors for constant data. Weighted frequency percentages were also computed for categorical variables. The TyG index’s quartile groupings were compared using either the Chi-squared or Kruskal-Wallis H test. The research used multivariate logistic regression models to examine the relationship between the TyG index and OA. In this study, three distinct models were employed. There were no modifications made to Model 1. Modifications were implemented in Model 2 concerning age, gender, and racial variables. In Model 3, adjustments were made for various demographic and health-related factors, including age, gender, race, education level, PIR, smoke status, alcohol consumption, hypertension, diabetes, moderate physical activity, BMI, HDL cholesterol, LDL cholesterol, insulin, HOMA-IR, ALT, AST, CRP, BUN, and UA. This study employed a weighted generalized additive model (GAM) regression using a penalized spline technique to evaluate the nonlinear relationship between the TyG index and OA. To better evaluate the nonlinear relationship between the TyG index and OA, threshold effect analysis was conducted. Moreover, a stratified multivariate regression analysis was conducted to perform subgroup analysis according to age, gender, BMI, smoking status, and diabetes. Furthermore, the incorporation of an interaction term facilitated the assessment of potential differences in relationships across subgroups. A *p*-value equal to or less than 0.05 was deemed statistically significant. The analyses were conducted using R version 3.4.3 (The R Foundation, http://www.R-project.org) and Empower software (X&Y Solutions, Inc., Boston, MA, USA).

## Results

### Baseline characteristics of participants

Table 1 presents the weighted demographic baseline characteristics of the people involved in the study. The study comprised a sample size of 25,514 individuals, with 49.06% male and 50.94% female. The participants had an average age of 48.05 ± 17.23 years. The average value of the TyG index was found to be 8.48 ± 0.65. The TyG index values for quartiles 1 to 4 were ≤ 8.02, 8.02–8.47, 8.48–8.93, and ≥ 8.93. The total average prevalence of OA was found to be 14.37%. When examining the data by quartiles, the prevalence rates were 9.05% for Quartile 1, 12.76% for Quartile 2, 18.25% for Quartile 3, and 17.66% for Quartile 4.

**Table 1:** Baseline characteristics of participants, weighted

Variables	Quartiles of TyG				*P* value
	Q1	Q2	Q3	Q4	
*Age [year]*	40.67 ± 16.67	48.49 ± 17.62	51.09 ± 16.41	52.24 ± 15.72	**< 0.0001**
*Gender (%)*					**< 0.0001**
Male	41.18	47.65	51.87	55.91	
Female	58.82	52.35	48.13	44.09	
*PIR (%)*	3.19 ± 1.54	3.08 ± 1.56	3.05 ± 1.60	2.97 ± 1.51	**0.0163**
*Race (%)*					**< 0.0001**
Mexican American	7.09	7.56	10.03	11.63	
Other Hispanic	5.53	7.77	8.42	6.98	
Non-Hispanic white	61.74	62.08	62.13	64.69	
Non-Hispanic black	17.41	11.98	8.45	5.04	
Other races	8.23	10.61	10.97	11.66	
*Education level (%)*					**0.0070**
Under high school	14.97	19.03	19.90	19.38	
High school or equivalent	22.36	25.40	22.79	25.34	
Above high school	62.68	55.57	57.31	55.28	
*BMI (%)*					**< 0.0001**
Normal (BMI < 25 kg/m^2^)	50.54	26.82	17.83	10.94	
Overweight (25 ≤ BMI ˂30 kg/m^2^)	29.55	37.25	35.51	31.84	
Obese (BMI ≥ 30 kg/m^2^)	19.91	35.93	46.66	57.22	
*Cigarette Use (%)*					**< 0.0001**
Yes (Smoked ≥ 100 cigarettes in life)	41.05	39.80	44.23	50.06	
No (Smoked < 100 cigarettes in life)	58.95	60.20	55.77	49.94	
*Alcohol Use (%)*					**< 0.0001**
Yes (drink at least once a month)	55.80	53.74	49.47	44.22	
No (drink no more than once a month)	36.10	35.55	39.90	44.05	
*Hypertension (%)*					**< 0.0001**
Yes	17.57	27.17	38.49	45.93	
No	82.43	72.83	61.51	54.07	
*Diabetes (%)*					
Yes	2.07	5.21	12.09	27.06	**< 0.0001**
No	97.93	94.79	87.91	72.94	
*Moderate physical activity (%)*					**0.0002**
Yes	76.12	70.79	69.00	67.85	
No	23.88	29.21	31.00	32.15	
*Insulin ((µU/L)*	7.86 ± 6.34	11.30 ± 10.35	16.34 ± 29.90	21.24 ± 30.46	**< 0.0001**
*HOMA-IR*	1.93 ± 1.72	2.96 ± 3.25	4.63 ± 8.69	7.49 ± 15.84	**< 0.0001**
*CRP [mg/L]*	2.45 ± 5.24	4.08 ± 8.02	4.34 ± 6.97	4.64 ± 7.51	**< 0.0001**
*ALT [U/L]*	18.31 ± 14.12	20.53 ± 14.25	24.38 ± 18.68	26.50 ± 18.00	**< 0.0001**
*AST [U/L]*	20.71 ± 12.23	21.34 ± 10.68	22.49 ± 14.66	22.26 ± 10.70	**0.0037**
*BUN [[mg/dL]*	13.92 ± 4.94	14.41 ± 5.04	14.81 ± 5.45	15.82 ± 6.29	**< 0.0001**
*UA [mg/dL]*	4.87 ± 1.27	5.28 ± 1.25	5.65 ± 1.42	5.90 ± 1.45	**< 0.0001**
*HDL-cholesterol [mg/dL]*	63.01 ± 16.54	58.05 ± 15.83	50.54 ± 13.13	44.53 ± 10.54	**< 0.0001**
*LDL-cholesterol [mg/dL]*	95.53 ± 28.11	109.95 ± 32.16	116.46 ± 35.39	117.05 ± 39.72	**< 0.0001**
*TyG [g/cm* ^*2*^ *]*	7.70 ± 0.26	8.25 ± 0.13	8.68 ± 0.13	9.34 ± 0.36	**< 0.0001**
*Osteoarthritis (%)*	9.05	12.76	18.25	17.66	**< 0.0001**

Mean ± SD for continuous variables: the *P* value was calculated by the weighted linear regression model. (%) for categorical variables: the *P* value was calculated by the weighted chi-square test

PIR, Poverty-to-income ratio; BMI, body mass index; HOMA-IR: homeostatic model assessment of insulin resistance; CRP, C-Reactive Protein; ALT, alanine transaminase; AST, aspartate aminotransferase; BUN, blood urea nitrogen; UA, uric acid; HDL-cholesterol, high-Density Lipoprotein Cholesterol; LDL-cholesterol, low-Density Lipoprotein Cholesterol; TyG, Triglyceride–Glucose

### Higher TyG index is associated with a greater risk of OA

**Fig. 2:**
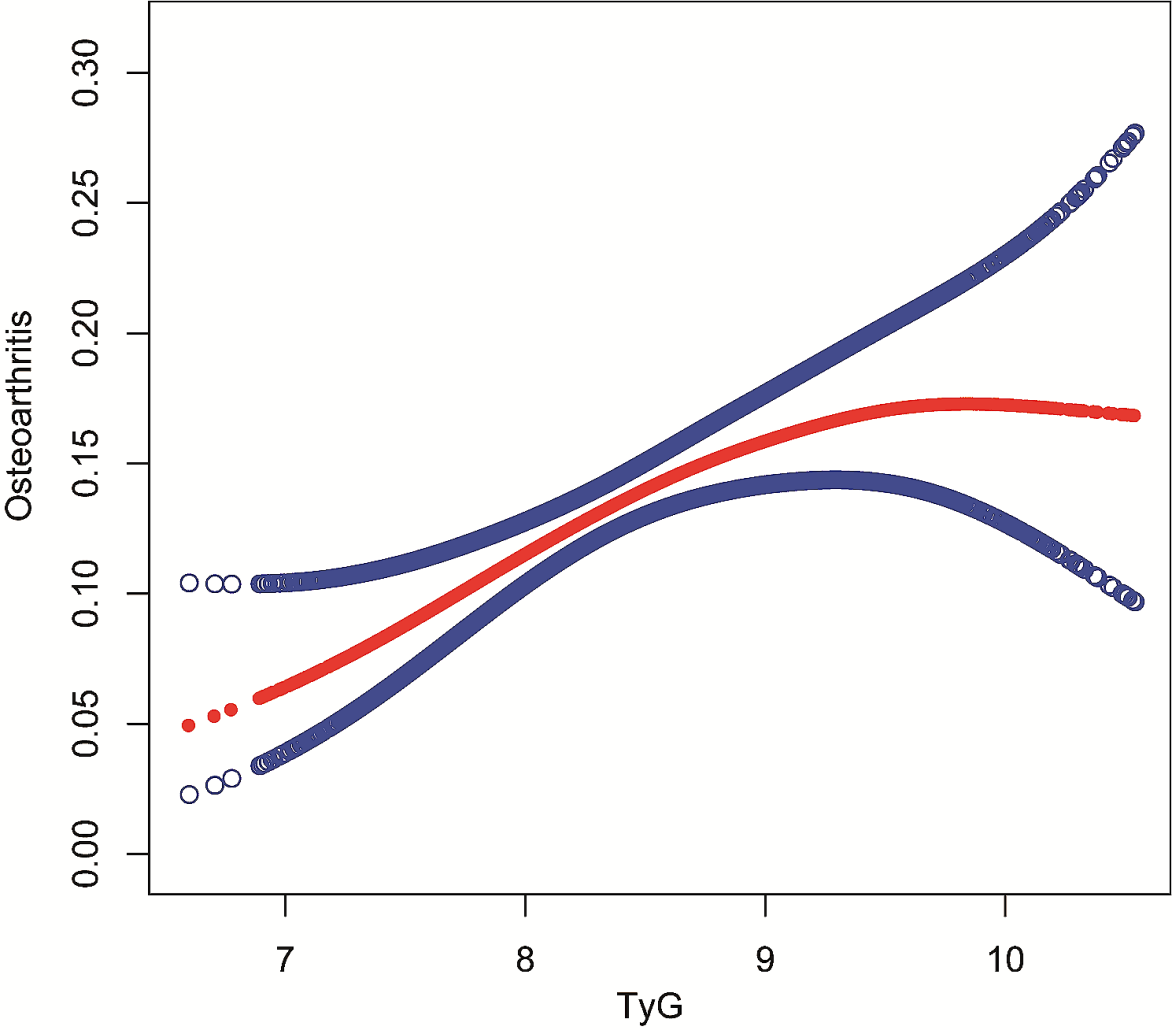
Association between TyG index and OA by the GAM. The solid red line represents the smooth curve fit between variables. Blue bands represent the 95% confidence bands derived from the fit

An affirmative correlation between the TyG index and OA was observed. In the third model, after adjusting for all relevant factors, the positive link remained consistent [OR = 7.34; 95% CI: 2.25, 23.93; *p* = 0.0010]. This suggests that for every unit rise in the TyG index, there is a 634% higher risk of developing OA. In addition, we discretized the TyG index, originally a continuous variable, into quartiles to do a sensitivity analysis. A statistically significant 69% higher probability of OA was reported in Quartile 4 compared to Quartile 1, representing the lowest TyG index quartile. Furthermore, the observed p trend suggests that the upward trend observed in all models is statistically significant, implying a propensity for the risk of OA to increase as the TyG index increases. The provided information is presented in Table 2. In order to investigate the nonlinear association between the TyG index and OA, weighted GAM and smooth curve fits were utilized. Our study’s findings revealed no discernible nonlinear association between the TyG index and OA, as depicted in Fig. 2. Moreover, Cox proportional hazards regression models were conducted. Based on threshold effect analysis, we identified the inflection points for OA as 9.67 (*P* values for log-likelihood ratio < 0.05) (Table 3).

**Table 2:** Association between TyG and OA

	OR (95% CI), *p* value		
	Model 1	Model 2	Model 3
Osteoarthritis			
TyG index	**9.52 (4.13, 21.92) < 0.0001**	**8.54 (3.41, 21.37) < 0.0001**	**7.34 (2.25, 23.93) 0.0010**
Categories			
Quartile 1	Reference	Reference	Reference
Quartile 2	1.26 (0.94, 1.68) 0.1217	1.15 (0.85, 1.56) 0.3523	1.12 (0.82, 1.54) 0.4760
Quartile 3	**1.76 (1.34, 2.32) < 0.0001**	**1.60 (1.20, 2.14) 0.0015**	**1.62 (1.17, 2.25) 0.0036**
Quartile 4	**1.93 (1.47, 2.54) < 0.0001**	**1.76 (1.31, 2.35) 0.0002**	**1.69 (1.18, 2.42) 0.0039**
P for trend	**9.52 (4.13, 21.92) < 0.0001**	**1.49 (1.24, 1.79) < 0.0001**	**1.45 (1.16, 1.83) 0.0014**

Data are presented as ORs, 95% CIs, and *P*-value

Model 1: adjusted for nothing

Model 2: adjusted for age, gender, and race

Model 3: adjusted for age, gender, race, education level, PIR, smoke status, alcohol consumption, hypertension, diabetes, moderate physical activity, BMI, HDL cholesterol, LDL cholesterol, insulin, HOMA-IR, ALT, AST, CRP, BUN, and UA

ALT: alanine transaminase; AST: aspartate aminotransferase; BMI: body mass index; BUN: blood urea nitrogen; CI: confidence interval; CRP: C-Reactive Protein; HDL cholesterol: high-Density Lipoprotein Cholesterol; HOMA-IR, homeostatic model assessment of insulin resistance; LDL cholesterol: low-Density Lipoprotein Cholesterol; OR: odds ratio; PIR:Poverty-to-income ratio; TyG: Triglyceride–Glucose; UA; uric acid

**Table 3:** Threshold effect analysis of TyG index and OA

Osteoarthritis	OR (95% CI)	*p* value
TyG index		
Inflection point		9.67
TyG index < Inflection point	1.54 (1.24, 1.91)	**0.0001**
TyG index > Inflection point	0.28 (0.07, 1.14)	0.0746
Log-likelihood ratio		**0.015**

Data are presented as OR, 95% CI, and *p* value

Model 1: adjusted for nothing

Model 2: adjusted for age, gender, and race

Model 3: adjusted for age, gender, race, education level, PIR, smoke status, alcohol consumption, hypertension, diabetes, moderate physical activity, BMI, HDL cholesterol, LDL cholesterol, insulin, HOMA-IR, ALT, AST, CRP, BUN, and UA

ALT: alanine transaminase; AST: aspartate aminotransferase; BMI: body mass index; BUN: blood urea nitrogen; CI: confidence interval; CRP: C-Reactive Protein; HDL cholesterol: high-Density Lipoprotein Cholesterol; HOMA-IR, homeostatic model assessment of insulin resistance; LDL cholesterol: low-Density Lipoprotein Cholesterol; OR: odds ratio; PIR:Poverty-to-income ratio; TyG: Triglyceride–Glucose; UA; uric acid

### Subgroup analysis

A subgroup analysis assessed the strength and reliability of the association between the TyG index and OA. The study examined the potential effects of age, gender, BMI, smoking status, and diabetes on the observed interactions. Nevertheless, our analysis did not find any statistically significant association between age, gender, BMI, and smoking status concerning this association. This suggests that the factors mentioned above do not significantly impact the observed relationship (all p for interaction > 0.05). Our study’s findings indicate a consistent positive correlation between the TyG index and OA across many groups, regardless of age, gender, BMI, smoking status, and diabetes. Interestingly, the participants without diabetes showed a stronger positive correlation between the TyG index and OA (p for interaction < 0.05). However, it is important to note that the NHANES data did not explicitly distinguish between T1MD and T2MD in its research design. Therefore, we could not run the subgroup analysis stratified by T1MD and T2MD further. This suggests that the relationship between these variables may be applicable in various demographic contexts, as seen in Table 4.

**Table 4:** Subgroup analysis

	Osteoarthritis	
	OR (95% CI), *p* value	P for interaction
*Stratified by gender*		0.4823
Male	1.28 (0.95, 1.71) 0.0989	
Female	1.46 (1.13, 1.88) 0.0036	
*Stratified by age*		0.2983
Age < 65	1.47 (1.16, 1.87) 0.0015	
Age ≥ 65	1.21 (0.89, 1.65) 0.2287	
*Stratified by BMI*		0.0923
Normal (BMI < 25 kg/m^2^)	2.12 (1.28, 3.52) 0.0037	
Overweight (25 ≤ BMI ˂30 kg/m^2^)	1.38 (0.99, 1.92) 0.0574	
Obese (BMI ≥ 30 kg/m^2^)	1.24 (0.94, 1.64) 0.1330	
*Stratified by smoking*		0.8307
Yes	1.34 (1.04, 1.72) 0.0258	
No	1.38 (1.08, 1.77) 0.0113	
*Stratified by diabetes*		**0.0465**
Yes	1.02 (0.69, 1.51) 0.9112	
No	**1.63 (1.28, 2.08) < 0.0001**	

Model 1 was adjusted for no covariates;

Model 2 was adjusted for age, gender, and race;

Model 3 was adjusted for all covariates except effect modifier

OR, odds ratio; 95% CI, 95% confidence interval

## Discussion

Our results show a statistically significant independent connection between raised TyG index levels and a higher likelihood of OA in this cross-sectional investigation, which included 25,514 participants. The subgroups stratified by age, gender, BMI, smoking status, and diabetes showed similarity in the detected link. Starting IR treatment and management earlier in life may have positive effects on reducing or preventing the occurrence of OA.

This is the first investigation of the relationship between the TyG index and OA. Numerous additional clinicopathological variables have been linked to OA in other research. Chen et al. [20] found a correlation between the frequency of OA and the levels of thyroid hormone sensitivity (central and peripheral). Lower blood 25-(OH)D levels were substantially and non-linearly linked to more significant risks of all-cause and cardiovascular diseases mortality in American people with OA, according to Xiao et al. [21]. Biological age, phenotypic age, and telomere length were revealed to have substantial mediating effects on the association of metals with OA risk by Chen et al. [22]. It has been shown in earlier research that inflammation and the TyG index are highly related. Li et al. [23] demonstrated that higher levels of inflammation and the TyG index, both individually and in combination, increased the risk of colorectal cancer. Moreover, Yan et al. [24] elucidated that among Americans under 60 with average weight and no diabetes, the TyG index was positively linked with arthritis. A combined predictor to represent innate and adaptive immunity in response to cancer, the systemic inflammatory response index (SIRI) was developed initially [25]. SIRI is a newly discovered biomarker for OA disease activity assessment [26]. The NF-κB and NLRP3 signaling pathways collaborate to support P2 × 7-induced chondrocyte extracellular matrix breakdown and pyroptotic inflammation, according to Li et al. [27]. In addition, Philpott et al. [28] showed how cannabidiol might delay the onset of chronic joint pain by inhibiting early OA-related inflammation. OA is a disease of the whole joint, affecting the synovium, tendons, muscles, ligaments, subchondral bone, adipose tissue, and articular cartilage. It may even be a systemic illness, with inflammation playing a crucial part in the interaction of the joint tissues [29]. Greene et al. [4] suggested that “inflame-aging”—or age-associated inflammation—and the onset of OA may be connected. Furthermore, the study conducted by Jin et al. [30] showed that limonin can inhibit NF-κB activation in chondrocytes. This inhibition occurs via the activation of the Nrf2/HO-1 cascade, significantly reducing the inflammatory response and catabolic processes induced by IL-1β. It has also been claimed that inflammation is favorably related to IR, and in TNF-treated Gulo-/- mice, Qing et al. [31] revealed that vitamin C deficiency worsens IR, which may partly be due to the loss of the anti-inflammation effect. Protein tyrosine phosphatase 1B (PTP1B) is a regulatory enzyme that catalyzes the removal of phosphate groups from phosphoserine residues found in insulin receptor and receptor substrate proteins. Additionally, it has been shown that the expression of PTP1B is increased in response to the pro-inflammatory cytokine TNF-α [32]. NF-κB activation via TLR4/MD-2 causes the production of NLRP3 and IL-1β and IL-18 precursors. When NLRP3 recognizes different DAMPs, it oligomerizes, activating caspase-1 and causing mature IL-1β and IL-18 to be produced. By serine phosphorylating IRS1/2, secreted IL-1 suppresses insulin receptor signaling and activates MAPKs via the IL-1 receptor (IL-1R) [33, 34]. In addition, obesity causes IR and macrophage infiltration in adipose tissue, the liver, and skeletal muscle [35]. TyG index has been considered a credible indication of IR, and as inflammation has been linked to both IR and OA, which may be positively correlated.

It needs to be clarified what causes the TyG index to be correlated with OA. Rosa et al. [36] have reported that chondrocytes express functional insulin receptors in human adults. In individuals with OA, chondrocytes have a diminished ability to enhance glucose transport in response to normal physiological levels of insulin. This impairment in glucose transport may harm energy production and the plastic functions of chondrocytes, including the synthesis of glucosaminoglycans. Ultimately, this can lead to chondrocyte damage and the progression of OA. Furthermore, studies have demonstrated that visceral fat, which serves as a substantial source of pro-inflammatory cytokines, can contribute to low-grade chronic metabolic inflammation. This inflammation can potentially lead to joint structural damage and is often associated with IR and T2DM [37]. Moreover, IR is crucial in controlling metabolic syndrome, which raises the risk of OA by maintaining joint inflammation [38]. The impairment of insulin’s ability to suppress the production of inflammatory and catabolic mediators responsible for OA would be diminished with the onset of IR in individuals with obesity. Insulin has a pivotal role as a significant modulator of synovial inflammation and catabolism. According to a publication, obese OA patients with T2DM have synovium that develops IR [16]. A greater TyG index is linked to a greater risk of OA, which may be explained by the TyG index’s positive correlation with the IR level.

Previous epidemiological research showed that OA risk factors included obesity, smoking, hypertension, and diabetes. Jiang et al. [39] discovered that females were substantially more likely than males to have KOA, with a 5-unit rise in BMI being linked to a 35% greater risk of the condition (p˂0.05). The pathophysiology of OA has previously been linked to vascular dysfunction [40]. Ching et al. [41] revealed that hypertension’s biophysical and biochemical effects on the synovium, subchondral bone, and chondrocytes disrupt joint homeostasis. They may be a factor in the development of OA. Smokers are more likely to develop spinal OA and have more intense and long-lasting pain than non-smokers, according to research by Felson et al. [42]. Thomas Rehling et al. [43] found that OA prevalence was strongly correlated with diabetes [1.3 (1.2–1.4), p˂0.001]. Our findings of the subgroup analysis show that, in line with previous research, the positive correlation was constant in subgroups stratified by gender, age, BMI, smoking, and diabetes. Furthermore, we found no dependency on BMI, smoking status, or diabetes for this connection (all p for interaction > 0.05), indicating that this positive correlation may be suitable for many demographic contexts. However, there existed a higher positive association between TyG index and OA in the individuals without diabetes (p for interaction < 0.05).

Current research has several advantages. The NHANES data were used in our investigation, and the appropriate NHANES sample weights were considered while performing the analyses. In addition, we used covariate modifications to lessen the impact of confounding variables, improving the dependability of our results and allowing them to be applied to a larger population. However, it is critical to recognize the constraints built into our research. Personal interviews were used to diagnose OA first, opening the door to the potential of recall bias. Lack of information on pertinent laboratory tests, such as anti-Cyclic Citrullinated Peptide antibodies and Rheumatoid Factor, for the diagnosis and differential diagnosis of OA is another limitation. Moreover, because of NAHNES database restrictions, individuals with OA did not have their occupation or comorbidities included as variables. Furthermore, it should be emphasized that the study design of the NHANES data did not specifically contain information about the differentiation between T1MD and T2MD diabetes. As a result, a more thorough investigation of this connection among subgroups stratified by T1MD and T2MD was not feasible. Furthermore, we chose to use only one imputation for managing the missing data, possibly affecting the accuracy of our findings, given that the missing values of the variables were randomly ignored and the sample size was large enough to draw a firm conclusion. The cross-sectional research design might be blamed for failing to prove a causal connection between TyG and OA.

## Conclusion

This research indicates that elevated TyG index values are associated with a higher likelihood of OA prevalence. Hence, we postulate that TyG may serve as a valuable predictor for OA, contributing to the early identification and prevention of OA. However, further extensive prospective studies are necessary to clarify the precise cause of this relationship.

## Data Availability

Publicly available datasets were analyzed in this study. Those data can be found here: www.cdc.gov/nchs/nhanes/.

## Abbreviations

ALT: Alanine transaminase
AST: Aspartate aminotransferase
BMI: Body mass index
BUN: Blood urea nitrogen
CDC: Centres for Disease Control
CI: Confidence interval
CRP: C-Reactive Protein
GAM: Generalized additive model
HDL cholesterol: High-Density Lipoprotein Cholesterol
HOMA-IR: Homeostatic model assessment of insulin resistance
IR: Insulin resistance
KOA: Knee osteoarthritis
LDL-cholesterol: Low-Density Lipoprotein Cholesterol
NCHS: National Centre for Health Statistics
NHANES: National Health and Nutrition Examination Survey
OA: Osteoarthritis
OR: Odds ratio
PIR: Poverty-to-income ratio
PTP1B: Protein tyrosine phosphatase 1B
SIRI: Systemic inflammatory response index
T1DM: Type 1 diabetes mellitus
T2DM: Type 2 diabetes mellitus
TyG: Triglyceride Glucose
UA: Uric acid
